# A time-dependent genome-wide SNP-SNP interaction analysis of chicken body weight

**DOI:** 10.1186/s12864-019-6132-0

**Published:** 2019-10-23

**Authors:** Fang-Ge Li, Hui Li

**Affiliations:** 1 College of Science, Northeast Agricultural University, Harbin, 150030 People’s Republic of China; 2 Key Laboratory of Chicken Genetics and Breeding, Ministry of Agriculture and Rural Affairs, Harbin, 150030 People’s Republic of China; 3 Key Laboratory of Animal Genetics, Breeding and Reproduction, Education Department of Heilongjiang Province, Harbin, 150030 People’s Republic of China; 4 College of Animal Science and Technology, Northeast Agricultural University, Harbin, 150030 People’s Republic of China

## Abstract

**Background:**

The important property of the quantitative traits of model organisms is time-dependent. However, the methodology for investigating the genetic interaction network over time is still lacking. Our study aims to provide insights into the mechanistic basis of epistatic interactions affecting the phenotypes of model organisms.

**Results:**

We performed an exhaustive genome-wide search for significant SNP-SNP interactions associated with male birds’ body weight (BW) (*n* = 475) at multiple time points (day of hatch (BW0) and 1, 3, 5, and 7 weeks (BW1, BW3, BW5, and BW7)). Statistical analysis detected 67, four, and two significant SNP pairs associated with BW0, BW1, and BW3, respectively, with a significance threshold at 8.67 × 10^− 12^ (Bonferroni-adjusted: 1%). Meanwhile, no significant SNP pairs associated with BW5 and BW7 were found. The SNP-SNP interaction networks of BW0, BW1, and BW3 were built and annotated.

**Conclusions:**

With strong annotated information and a strict significant threshold, SNP-SNP interactions underpinned the gene-gene interactions that might occur between chromosomes or within the same chromosome. Comparing and combing the networks, the results indicated that the genetic network for chicken body weight was dynamic and time-dependent.

## Background

Epistatic interactions (non-linear interactions between segregating loci) are gaining attention in contemporary biology, yet their role in the genetic architecture of quantitative traits is still obscure and controversial. Studies on fruit fly (*Drosophila melanogaster*), yeast (*Saccharomyces cerevisiae*), mouse (*Mus musculus*), thale cress (*Arabidopsis thaliana*), maize (*Zea mays*), and human (*Homo sapiens*) demonstrate that epistasis is pervasive and is an important factor in determining the variation of quantitative phenotypes [1–4]. On the other hand, in the past 15 years, thousands of genome-wide association studies have reported numerous single SNP loci that exhibit significant additive effects; however, especially for quantitative traits and complex diseases, the results were challenged for missing heritability. A typical example is human height, which is a classic quantitative trait with an estimated heritability of about 80%, and has been associated with more than 700 SNP loci, which, however, explain only about 20% of heritability [5, 6]. Geneticists have postulated that identifying epistatic interactions between SNP loci would be a reasonable way to explain heritable variance [7]. Some studies have clarified this idea, including the study by Zuk et al. that demonstrated that a large part of the missing heritability of Crohn’s disease could be due to genetic interactions [8].

Carlborg et al. revealed that an apparently major locus for chicken growth belonged to a genetic network of four interacting loci, which indicates that epistatic interactions between genes (or quantitative trait loci) were important for quantitative traits in chicken [9]. Furthermore, our previous studies also detected epistatic interactions and demonstrated that they could affect the variation in chicken phenotypes [10–12].

In the current study, we focused on the body weight of chickens whose phenotypic data could be analyzed as a series of data points (i.e. time series). The chickens’ lifespan was divided into four equal periods of 2 weeks. The body weight at five time points was selected as a phenotypic value. Significant SNP-SNP interactions associated with the body weights at different weeks (BW0, BW1, BW3, BW5, and BW7) were detected with an exhaustive genome-wide test. Next, the SNP-SNP interaction networks were built and annotated. Our results provide further insight into the genetic network that controls body weight in chickens.

## Results

### SNP genotyping and phenotypic values

After quality control, the following was included in this study: a total of 48,152 SNPs on 28 autosomes, the Z chromosome, linkage groups, and 672 SNPs not assigned to any chromosomes in chickens (Table 1). Finally, 48,034 SNPs with chromosome position information were filtered for the interaction analysis.

**Table 1:** Summary of genome-wide markers

GGA^1^	SNPs’ number	GGA length (Mb)	Mean distance (kb)	GGA	SNPs’ number	GGA length (Mb)	Mean distance (kb)
1	7538	200.95	26.66	17	922	10.61	11.51
2	5652	154.79	27.39	18	917	10.89	11.87
3	4322	113.65	26.30	19	880	9.90	11.25
4	3518	94.16	26.77	20	1574	13.92	8.84
5	2295	62.23	27.11	21	796	6.95	8.73
6	1814	35.84	19.76	22	327	3.89	11.90
7	1907	38.17	20.01	23	643	6.02	9.37
8	1486	30.62	20.61	24	758	6.37	8.40
9	1240	24.02	19.37	25	181	2.02	11.17
10	1379	22.42	16.26	26	670	5.03	7.51
11	1312	21.87	16.67	27	506	4.84	9.56
12	1425	20.46	14.36	28	607	4.47	7.37
13	1204	18.32	15.21	LGE22	115	0.88	7.67
14	1062	15.76	14.84	LEG64	3	0.02	6.80
15	1082	12.93	11.95	Z	2001	74.59	37.28
16	16	0.42	26.12	UN^a^	672	/	/

1GGA is an abbreviation for *Gallus gallus*

^a^These SNPs were not assigned to any chromosomes

Phenotypic descriptive statistics for body weight are listed in Table 2. The body weights exhibited no significant differences between the lean and fat lines, so we mixed the two lines into one group for the interaction tests. The correlation coefficients between the different body weights of different weeks were calculated in the combined population (Table 3). The correlation coefficient between BW0-BW7 was near zero (0.015), indicating that BW0 and BW7 are uncorrelated, which was the minimum in Table 3. The correlation coefficients between BW0-BW1, BW1-BW3, BW3-BW5, and BW5-BW7 steadily increased and exhibited high values between 0.65–0.69.

**Table 2:** The Mean ± Standard deviation (SD) of the body weight in lean and fat lines, respectively, and in the combined population

Traits	Combined population (475 birds)	Lean line (203 birds)	Fat line (272 birds)
BW0 (g)	44.76 ± 3.39	44.83 ± 2.79	44.70 ± 3.78
BW1 (g)	121.97 ± 12.34	121.05 ± 12.80	122.68 ± 11.95
BW3 (g)	615.22 ± 65.97	617.35 ± 71.98	613.65 ± 61.23
BW5 (g)	1491.19 ± 142.53	1487.53 ± 159.13	1493.91 ± 129.10
BW7 (g)	2400.97 ± 221.65	2419.53 ± 246.45	2387.11 ± 200.51

**Table 3:** The correlation coefficient between body weights in the combined population

	BW 0	BW 1	BW 3	BW 5	BW 7
BW 0	1	0.357	1	0.037	0.015
BW 1		1	0.590	0.363	0.283
BW 3			1	0.694	0.436
BW 5				1	0.646
BW 7					1

### Epistatic analysis of body weight

MatrixEpistasis [13] is an ultrafast method that performs an exhaustive epistatic scanning for quantitative traits with covariate adjustment, and was applied to the interaction tests. With the significance threshold of 8.67 × 10^− 12^ (Bonferroni-adjusted: 1%), 67 (Table 4), four (Table 5), and two (Table 6) statistically significant SNP pairs associated with BW0, BW1, and BW3 were detected, respectively, with no replicated significant pairs. There were no SNP pairs with a *p*-value smaller than the threshold in BW5 and BW7.

**Table 4:** Genome-wide significant pairwise epistatic interactive SNP pairs for BW0

GGAF^a^	SNP1 name	GGAS^a^	SNP2 name	*P*-value
chr1	Gga_rs10727935	chr13	GgaluGA096558	8.36E-12
chr1	GgaluGA010224	chr4	Gga_rs14493884	3.35E-12
chr1	GgaluGA016211	chr6	Gga_rs14588369	5.14E-12
chr1	GgaluGA016211	chr7	Gga_rs14614638	8.22E-12
chr1	Gga_rs13982417	chrz	Gga_rs15249625	9.59E-14
chr11	GGaluGA078115	chrz	Gga_rs14759127	3.84E-12
chr12	Gga_rs14031249	chr19	GgaluGA124944	2.99E-12
chr12	GgaluGA081258	chr19	GgaluGA125308	3.89E-12
chr14	Gga_rs15719971	chr20	Gga_rs14277625	3.21E-12
chr19	Gga_rs10730456	chrz	Gga_rs16774940	3.15E-14
chr19	Gga_rs10730456	chrz	Gga_rs16774954	3.15E-14
chr19	Gga_rs10730456	chrz	Gga_rs14775753	1.06E-12
chr19	Gga_rs14118327	chrz	Gga_rs16774940	1.68E-13
chr19	Gga_rs14118327	chrz	Gga_rs16774954	1.68E-13
chr19	Gga_rs14118327	chrz	Gga_rs14775753	6.41E-12
chr19	Gga_rs15045504	chrz	Gga_rs16774954	6.81E-12
chr19	Gga_rs15045504	chrz	Gga_rs16774940	6.81E-12
chr19	Gga_rs15045732	chrz	Gga_rs16774940	7.62E-14
chr19	Gga_rs15045732	chrz	Gga_rs16774954	7.62E-14
chr19	Gga_rs15045732	chrz	Gga_rs14775753	3.04E-12
chr19	Gga_rs15048206	chrz	Gga_rs16774954	2.53E-13
chr19	Gga_rs15048206	chrz	Gga_rs16774940	2.53E-13
chr19	Gga_rs15048206	chrz	Gga_rs14775753	7.33E-12
chr19	Gga_rs15048223	chrz	Gga_rs16774940	5.90E-13
chr19	Gga_rs15048223	chrz	Gga_rs16774954	5.90E-13
chr19	GgaluGA126270	chrz	Gga_rs16774954	5.78E-12
chr19	GgaluGA126270	chrz	Gga_rs16774940	5.78E-12
chr2	GgaluGA160608	chr6	GgaluGA295929	7.73E-12
chr2	Gga_rs14135538	chrz	Gga_rs14755141	2.94E-12
chr2	Gga_rs14135538	chrz	Gga_rs16101791	4.79E-12
chr2	Gga_rs14184594	chrz	Gga_rs16106712	6.80E-13
chr2	Gga_rs14184594	chrz	Gga_rs16065879	7.44E-13
chr2	Gga_rs14184594	chrz	Gga_rs16091913	7.44E-13
chr2	Gga_rs14184594	chrz	Gga_rs16106257	7.44E-13
chr2	Gga_rs14184594	chrz	Gga_rs14738375	7.44E-13
chr2	Gga_rs14184594	chrz	Gga_rs16071074	7.44E-13
chr2	Gga_rs14184594	chrz	Gga_rs16091907	7.44E-13
chr2	Gga_rs14184594	chrz	Gga_rs16106786	7.44E-13
chr2	Gga_rs14184594	chrz	Gga_rs16776264	1.58E-12
chr24	GgaluGA193154	chrz	Gga_rs14753903	2.79E-12
chr27	Gga_rs16208036	chrz	Gga_rs14762941	3.30E-13
chr27	Gga_rs16208036	chrz	Gga_rs16108466	6.00E-13
chr27	GgaluGA199670	chrz	GgaluGA349792	7.85E-13
chr3	Gga_rs14082553	chr19	GgaluGA126270	9.81E-14
chr3	GgaluGA210739	chr19	Gga_rs14119969	5.99E-12
chr3	Gga_rs14323198	chr3	Gga_rs14394679	4.13E-12
chr3	Gga_rs14323198	chr3	Gga_rs14395789	4.25E-12
chr3	Gga_rs15426103	chr3	Gga_rs14323198	7.57E-12
chr3	Gga_rs16320563	chr3	Gga_rs14323198	1.25E-12
chr3	Gga_rs14385387	chrz	GgaluGA349792	1.91E-12
chr4	GgaluGA267201	chr13	GgaluGA093775	3.69E-12
chr4	GgaluGA268612	chr4	Gga_rs16424343	4.73E-13
chr4	Gga_rs14490998	chr5	GgaluGA289789	4.32E-12
chr4	Gga_rs13665914	chr9	Gga_rs13608349	1.59E-12
chr4	Gga_rs15474576	chrz	Gga_rs16108466	2.69E-13
chr5	Gga_rs15736571	chrz	Gga_rs14767978	7.14E-12
chr7	Gga_rs10728585	chrz	Gga_rs14759127	2.29E-12
chr7	Gga_rs15871969	chrz	Gga_rs14759127	1.08E-13
chr7	Gga_rs15871969	chrz	Gga_rs14759170	2.49E-12
chrz	Gga_rs13816749	chrz	GgaluGA350520	2.63E-12
chrz	Gga_rs14691748	chrz	GgaluGA350520	7.93E-13
chrz	Gga_rs14762941	chrz	Gga_rs16763798	8.37E-12
chrz	Gga_rs16080645	chrz	GgaluGA350520	2.63E-12
chrz	Gga_rs16685135	chrz	GgaluGA350520	2.63E-12
chrz	Gga_rs16763798	chrz	Gga_rs16108466	1.92E-12
chrz	Gga_rs16764173	chrz	Gga_rs16763798	7.77E-12
chrz	Gga_rs16764637	chrz	Gga_rs16764173	1.60E-12

^a^GGAF = The first chromosome in the pairwise epistasis analysis; GGAS = The second chromosome in the pairwise epistasis analysis; *P*-value = *P*-value of the effect being tested

**Table 5:** Genome-wide significant pairwise epistatic interactive SNP pairs for BW1

GGAF^a^	SNP1 name	GGAS^a^	SNP2 name	*P*-value
chr1	GgaluGA061615	chr4	Gga_rs14491923	2.39E-12
chr2	GgaluGA136764	chr3	GgaluGA211595	3.79E-13
chr2	GgaluGA133839	chr5	Gga_rs13756660	2.41E-12
chr2	GgaluGA133839	chr5	Gga_rs14515483	8.44E-12

^a^GGAF = The first chromosome in the pairwise epistasis analysis; GGAS = The second chromosome in the pairwise epistasis analysis; P-value = *P*-value of the effect being tested

**Table 6:** Genome-wide significant pairwise epistatic interactive SNP pairs for BW3

GGAF^a^	SNP1 name	GGAS^a^	SNP2 name	P-value
chr5	GgaluGA273676	chr11	Gga_rs14965049	2.80E-13
chr7	Gga_rs13598324	chr12	Gga_rs14045047	3.88E-12

^a^GGAF = The first chromosome in the pairwise epistasis analysis; GGAS = The second chromosome in the pairwise epistasis analysis; *P*-value = *P*-value of the effect being tested

### SNPs’ interaction networks

**Fig. 1:**
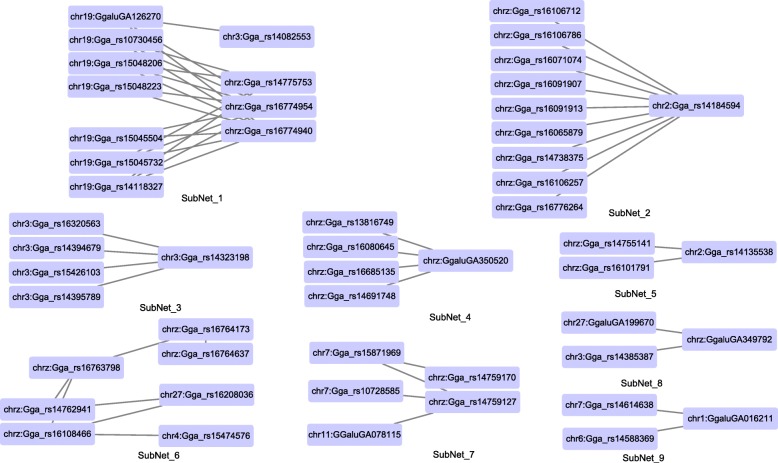
Epistatic SNP-SNP interaction network of birthday body weight (BW0) in NEAUHLF. One node represents one SNP whose name and chromosome number are shown in the rectangle. Significant SNP-SNP interactions were connected by the edge

The SNP-SNP interaction network of BW0 is constituted of separated subnets, and the subnets containing more than three nodes are shown in Fig. 1. The SNP epistatic interaction network is approximatively ‘small world’ and scale-free, both major topological features of interaction networks in biology. ‘Small world’ means shorter paths and independent subnets, resulting in dense local neighborhoods of genes that interact with each other [1]. The results of gene-gene interaction will be inferred in the next step. The scale-free property of networks implies that Gga_rs14184594 is the hub locus with the maximum degree.

### Annotation of SNP loci and SNP-SNP interaction networks

In total, 401 genes and 41 microRNAs were annotated to the BW0 network. Furthermore, 25 genes and one microRNA were annotated to the BW1 network and 19 genes and one microRNA to the BW3 network (Additional file 1: Table S1, Additional file 2: Table S2 and Additional file 3: Table S3).

The interaction network feature analysis was applied to the BW0 network, because the interaction network size and topology structure of BW1 and BW3 were small and apparent. The significant interaction SNP-SNP pairs contain 80 single SNPs in which 30 SNPs are located in the Z chromosome. Observing the annotation information indicated something interesting. Many SNPs from the same subnet are neighbors, concentrating in the same region. Therefore, we adjusted the spatial position of SNPs in Fig. 1, placing SNPs closer together if they were in the same region. All the subnets include SNPs from the same region, except SubNet_8 and SubNet_9. The phenomenon enhanced the reliability to infer that SNP-SNP interactions would be the result of gene-gene interactions in the correspond regions. For example, the cross lines in Subnet_1 accounted for the interactions between chr19: (3,823,581, 5,935,922) and chrz: (65,912,281, 67,063,604) and chr19: (1,728,331, 3,504,813) and chrz: (65,912,281, 67,063,604), which would be the signal of the gene set (*INIP*, *GNG10*, *SMC2*, *PTGR1*, *TXN*, *MUSK*, *LPAR1*) on the Z chromosome interacting with the gene set on the chromosome 19 (*CUX1, PRKRIP1, ORAI3* et al.). Furthermore, SubNet_2 claimed the gene set (*GLDC*, *TYRP1*, *MPDZ*, *NFIB*, *ZDHHC21*, *CER1*, *PSIP1*) on the Z chromosome interacting with the gene set (*IGFBP1*, *IGFBP3*, *TNS3*, *SLC12A7*) near the hub SNP on the chromosome 2. All the inferred gene set interactions are shown in Table 7. However, the annotation would generate a gene set in each region, thus we could conclude the interaction existing between gene sets, whereas the point-to-point interactive relationship could not be provided.

**Table 7:** Gene sets interaction inferred from the network of BW0

SubNet	Gene set A	Gene set B
1	*CUX1, PRKRIP1, ORAI3, RASA4, YWHAG, HSPB1, POR, RAD51D, RFFL, LIG3, CCLI5, CCL1, CCAH221, MRPS17, NIPSNAP2, PSPH, CCT6A, PHKG1, CHCHD2, VKORC1L1, GUSB, ASL2, ASL1, CRCP, KCTD7, NCBP3, CRK, YWHAE, MYO1C, INPP5K, SERPINF1, SMYD4, RPA1, DPH1, HIC1, POLDIP2, VTN, SLC46A1, ALDOC, TLCD1, FAM222B, ERAL1, FLOT2*	*INIP, GNG10, SMC2, PTGR1, TXN, MuSK, LPAR1*
1	*CUX1, PRKRIP1, ORAI3, RASA4, YWHAG, HSPB1, POR, RAD51D, RFFL, LIG3, CCLI5, CCL1, CCAH221, MRPS17, NIPSNAP2, PSPH, CCT6A, PHKG1, CHCHD2, VKORC1L1, GUSB, ASL2, ASL1, CRCP, KCTD7, NCBP3, CRK, YWHAE, MYO1C, INPP5K, SERPINF1, SMYD4, RPA1, DPH1, HIC1, POLDIP2, VTN, SLC46A1, ALDOC, TLCD1, FAM222B, ERAL1, FLOT2*	*DTD1, SEC23B, DZANK1, BIRC5, POLR3F, KAT14, MGME1, SNX5, RRBP1, DSTN, CST3, CST7, APMAP, TTBK1, SLC22A7, TTL, VSX1, ENTPD6, MAL, MRPS5, SLC8A1*
1	*CASTOR2, RCC1L, NCF1, RFC2, LAT2, EIF4H, UBE2G1, ATP2A3, P2RX1, MIS12, RABEP1*	*INIP, GNG10, SMC2, PTGR1, TXN, MuSK, LPAR1*
2	*GLDC, TYRP1, MPDZ, NFIB, ZDHHC21, CER1, PSIP1*	*IGFBP1, IGFBP3, TNS3, SLC12A7*
3	*MYOM2, CLN8, ERICH1, ACP1, TMEM18*	*GPATCH2, ESRRG*
4	*GLDC, TYRP1, MPDZ, NFIB, ZDHHC21, CER1, PSIP1*	*GLDC, TYRP1, MPDZ, NFIB, ZDHHC21, CER1, PSIP1*
5	*INSIG1, EN2, SHH, LMBR1, MNX1, UBE3C, DNAJB6*	*SLC30A5, CENPH, THBS4, MTX3*
6	*S100Z, F2RL1, IQGAP2, POLK, HMGCR, NSA2, UTP15, ANKRA2, FOXD1*	*ITGA1, PELO, FST*
6	*ITGA1, PELO, FST*	*NFIB, ZDHHC21, CER1, PSIP1*
6	*MRPL45, CBX1, NFE2L1, CDK5RAP3, LOC107055293, PCGF2, RPL23, LASP1, RPL19, ERBB2, IKZF3, ZPBP2, GSDMA, PSMD3, CSF3, MED24, THRA, RARA, TOP2A, IGFBP4, CCR7, SMARCE1, KRT222, KRT20*	*NFIB, ZDHHC21, CER1, PSIP1*
6	*BTK, TIMM8A, TAF7L, CENPI, DKC1, MPP1, RHOGL, NONO, GJB1, NLGN3, IL2RG, SNX12, LOC422214, HTR2C, IL13RA2, PLS3*	*NFIB, ZDHHC21, CER1, PSIP1*
7	*S100Z, F2RL1, IQGAP2, POLK, HMGCR, NSA2*	*SEC22A, ADCY5, HACD2, MYLK*
7	*METTL21A, CREB1, KLF7, ADAM23, EEF1B2, NDUFS1, NRP2*	*S100Z, F2RL1, IQGAP2, POLK, HMGCR, NSA2*
7	*S100Z, F2RL1, IQGAP2, POLK, HMGCR, NSA2*	*NUDT7*
8	*ACE, KCNH6, DCAF7, LIMD2, RNF113A, STRADA, DDX42, MYL4, CDC27, MAPT, ITGA3, DLX3, KAT7, SLC35B1, NGFR, MEOX1, PHB*	*POLK, HMGCR, NSA2, UTP15, ANKRA2, FOXD1*
8	*TMEM30A, COX7A2, COL12A1, SLC17A5, EEF1A1, MTO1*	*POLK, HMGCR, NSA2, UTP15, ANKRA2, FOXD1*
9	*ARL1, CNOT4, WDR91, PDE6H, ARHGDIB, MGP, OC3, ART4, HIST1H2B7, HIST1H46L2, HISTH2A4L1, HIST2H4B, HIST1H46, HIST1H2B7, HIST1H110, HIST1H46L2, HIST1H2B8, HIST2H3L, H2AFJ, HIST1H103, HIST1H2A4, HIST1H101, HIST1H111L, HIST1H2A4L3, HIST1H2B5, HIST1H3H, HIST1H111R, DDX47, HEBP1, FAM234B, EMP1*	*IFIH1, FAP, GCG, DPP4*
9	*ARL1, CNOT4, WDR91, PDE6H, ARHGDIB, MGP, OC3, ART4, HIST1H2B7, HIST1H46L2, HISTH2A4L1, HIST2H4B, HIST1H46, HIST1H2B7, HIST1H110, HIST1H46L2, HIST1H2B8, HIST2H3L, H2AFJ, HIST1H103, HIST1H2A4, HIST1H101, HIST1H111L, HIST1H2A4L3, HIST1H2B5, HIST1H3H, HIST1H111R, DDX47, HEBP1, FAM234B, EMP1*	*TECTB, ACSL5, VTI1A, TCF7L2, HABP2, DCLRE1A, NHLRC2*

The interaction effects could be detected in the same or different chromosomes. SubNet_3 and SubNet_4 indicated that the interaction effect could happen within the same chromosome. Furthermore, SNPs in SubNet_4 were all in the same region, neighborhood genes interacting with each other. Other SubNets all illustrated that the interaction effects were present in different chromosomes.

Eight gene ontology terms were significantly enriched, including chromosome, nucleus, phosphoprotein, acetylation, DNA-binding, nucleosome, nucleosome core, and histone H5 (Additional file 4: Table S4). Five pathways were significantly enriched, including the calcium signaling pathway, focal adhesion, ECM-receptor interaction, melanogenesis, and oocyte meiosis (Additional file 5: Table S5).

## Discussion

To our knowledge, this study used a novel approach by detecting the interaction effects that affect the quantitative traits of phenotype variation at multiple time points with SNP data. Based on the recognition that phenotypic data continuously changes, which is a key feature of quantitative traits, we assessed the similarity of the genetic network of quantitative traits at different periods. Many studies have evaluated interaction effects; however, no studies have assessed whether the genetic networks are time-dependent. Our study has demonstrated that genetic networks are time-dependent, contributing to our understanding of this field.

Chicken (*Gallus gallus*) is a vertebrate, a model organism, and agricultural species, and its body weight is a typical quantitative trait that can be easily measured. Broiler body weight’s heritability in males ranges from 0.29 to 0.37 [14, 15], a medium to high level. In the experimental population of this study, body weight’s heritability in males ranged from 0.28 to 0.53. Thus, broiler body weight is a suitable quantitative trait for detecting interaction effects and determining the features of genetic networks.

The male body weight data used in the study were derived from NEAUHLF, a broiler line. Although the resource population contains both the lean and fat lines, we decided to use all the samples in the current study. The decision was based on the selection of population, birds breeding, and phenotypic value’s statistical character. To be specific, the two lines had to come from the same grandsire line and be raised under the same environmental conditions. Body weights of male birds in the second hatch were considered. As a result, body weights did not exhibit significant differences between the lean and fat lines (Table 2). More importantly, larger sample numbers improve the interaction test power. We performed the test in the lean and fat lines separately, yet no significant SNP pairs were detected. Thus, the phenotypic values were not divided according to the line.

In the previous study, pair-wise interaction effects associated with important traits in chickens have been identified by the EPISNP3 module in epiSNP_v4.2_Windows software package [12, 16]. However, no significant epistatic interactions affecting body weight (BW1, BW3, BW5, and BW7) were detected. In the current study, we focused on bird’s body weight. From day of hatch to 7 weeks, the phenotypic data contained five time points, which were treated as time series data. Furthermore, the new method MatrixEpistasis, which can remove confounding bias through covariate adjustment, was used [13]. Population genomic stratification might occur in the long-term artificially selected population due to selection pressure. MatrixEpistasis can handle this bias and offers another advantage of ultra-computational speed, the critical factor for SNP-SNP interaction mapping at the genome-wide level. With the new method, we found some interesting results.

Testing multiple hypotheses caused that the significance threshold *p*-value (8.67 × 10^− 12^, Bonferroni-adjusted: 1%) was far smaller than 1%. The significant test results heavily depended on the arbitrary significance threshold. Although some effects were thus ignored, the strict threshold enhanced the confidence of our results. With the strict threshold, the detection results showed that the interaction effect was completely different at different time points. This suggests that the time point is an important factor in the quantitative trait interaction test. It is easy to determine the interaction effect on the day of hatch, whereas it is difficulty at 5 and 7 weeks. This demonstrates that the cooperation between genes is closer in the early weeks than later weeks. From the perspective of data driving, the correlations between the body weight at BW0 and other weeks were relatively small, which can partially explain the different results. Furthermore, the results indicate that the genetic regulation networks are different at different time points. Carlborg et al. [17] have found a similar conclusion in chicken.

Many SNP positions on the chromosome are neighboring in the SNP-SNP interaction network. We suggest it should be the signal of gene-gene interactions in the corresponding regions.

In this study, we detected 55 regions on 17 chromosomes. Based on the literature, some genes are associated with body weight; for example, *IGFBP1* is associated with body weight in the Jinghai Yellow chicken [18]. *MuSK* was abundantly expressed in the muscle of early-stage fowl embryos [19]. *ADCY5* is related to obesity in men and mice [20]. *PHKG1* is important in pig skeletal muscle [21]. We identified five pathways related to body weight. The focal adhesion signal pathway plays an important role in the development of chicken muscle [22]. The focal adhesion and ECM-receptor interaction signal pathways are related to intramuscular fat content [23]. In addition, numerous genes are associated with human complex diseases, such as *ATP2A3* [24], *ITGA1* [25], and *THBS4* [26].

Linkage disequilibrium and quantitative trait locus information were not introduced in this study, because linkage disequilibrium testing is not correlated with epistatic interaction tests and gene-gene results are usually more accurate than quantitative trait locus interactions. In fact, gene-gene interaction results were based on the gene sets, and the actual gene-gene interactions could be verified by biological experiments.

In the future, more research will be needed to better understand the genetic network of quantitative traits.

## Conclusions

The interaction effect is most active on the day of hatch, after which the effects decline, and by 5–7 weeks the effects are hardly detected. No significant SNP pairs recurred at different time points. Our study demonstrated that the genetic interaction network of chicken body weight is time-dependent and the epistatic interaction effect is dynamic. For the first three time points, the interaction networks indicated that SNP-SNP interactions were concentrated in some special regions on the chromosomes, which were the results of gene-gene interactions. To our knowledge, we are the first to describe and summarize the significant interaction effects that affect chicken body weight variation.

## Methods

### Experimental population

A total of 475 male chickens derived from the 11th generation population of the Northeast Agricultural University broiler lines divergently selected for abdominal fat content (NEAUHLF) were used in the study.

The G0 generation was selected in 1996 and came from the same grandsire line, which originated from the Arbor Acres broiler. According to their plasma very low-density lipoprotein (VLDL) concentration at 7 weeks of age, the G0 birds were divided into two lines, the lean line and the fat line. For each line, 25 half-sib families, with an average of 70 G1 offspring per family in two hatches, were produced by mating the G0 birds (one sire: four dams). From G1 to G11, the birds were raised in two hatches. Abdominal fat percentage (AFP = abdominal fat weight/body weight, measured at 7 weeks of age) of the male birds in the first hatch was used as the artificial selection criterion for NEAUHLF. The families’ sib birds, with lower (lean line) or higher (fat line) AFP than the population’s average value, were selected as candidates for breeding, taking into consideration the plasma VLDL concentration, the body weights of male birds in the second hatch, and the egg production of female birds in both hatches. G11 contained 203 chickens in the lean line and 272 chickens in the fat line.

All the birds were fed with a corn-soybean-based diet that met all National Research Council (NRC) requirements and were raised under the same environmental conditions with free access to feed and water. From hatch to 3 weeks of age, the birds received a starter feed (3000 kal ME = kg and 210 g = kg CP). From 4 weeks of age to slaughter, the birds were fed a grower diet (3100 kal ME = kg and 190 g = kg CP). The birds were weighed at day of hatch and at 1, 3, 5, and 7 weeks of age [11, 12].

### SNP genotyping and phenotypic values

Genotyping was carried out using chicken 60 K SNP chip (57,636 SNPs) manufactured by the Illumina Inc. (San Diego, CA). Monomorphic or minor allele frequencies (< 5%) loci were filtered out. Individuals whose missing SNP genotypes were ≥ 5% were removed. After quality control, SNPs with chromosome position information were selected for the interaction analysis. Phenotypic values were analyzed with descriptive statistics. The correlation coefficients of body weight among different weeks age were calculated in the combined population.

### Genome-wide pairwise interaction analysis

The method for detecting the interaction effect was implemented in R and is available at https://github.com/fanglab/MatrixEpistasis. The statistical model was:

$$ p=\alpha +{\beta}_1{G}_{.s}+{\beta}_2{G}_{.t}+{\beta}_3{G}_{.s}{G}_{.t}+{\sum}_v{\gamma}_v{C}_{.v}+\varepsilon $$

Where *α* is the overall mean of the quantitative phenotype; *β*_1_ / *β*_2_, *β*_3_ and *γ*_*v*_ are the regression coefficients for the main genetic additive effect, interaction effect, and covariates, respectively, and *ε* is a normal variable with zero mean and *ξ*^2^ variance [13]. In this model, the phenotype has both main genetic additive effects and covariates adjusted, and the size of the interaction effect is the regression coefficient *β*_3_. Therefore, the hypotheses are H_0_: *β*_3_ = 0 and H_1_: *β*_3_ ≠ 0; the tests of interaction correspond to testing whether the regression coefficient *β*_3_ equals zero or not.

MatrixEpistasis [13] was used to identify pairwise (two-dimension, SNP-SNP) significant epistatic interaction effects affecting body weight variation. The significance threshold was set at 8.67 × 10^− 12^ (Bonferroni-adjusted: 1%).

### SNP-SNP interaction network

The plots illustrating the SNP-SNP interaction networks with the significant epistatic effects for chicken body weight were drawn with the Cytoscape 3.7.0 software package [14]. The detailed network analysis was mainly applied to the BW0 network, and the BW1 and BW3 networks were sketched, because they were obvious and apparent.

### Annotation of the SNP-SNP interaction networks

For annotating genes to the interaction networks, a 1 Mb length region was designated for each SNP, 0.5 Mb upstream and 0.5 Mb downstream. The regions were merged if the distance between SNPs was < 1 Mb. Genes overlapping the regions were retrieved from UCSC (https://genome.ucsc.edu/) (Galgal5). Genes in the same region were put together (the so-called “gene set”). Gene interactions in gene sets were deduced if the significant SNP pairs were located in the corresponding regions.

Functional annotation of genes was performed by DAVID bioinformatics resources 6.8 (https://david.ncifcrf.gov/home.jsp) for gene ontology terms. KOBAS2.0 [27] was used for Kyoto Encyclopedia of Genes and Genomes (KEGG) pathway analysis. The significance threshold was set to the corrected *p*-value < 0.05.

## Supplementary information

**Additional file 1: Table S1**. Annotation information of BW0.

**Additional file 2: Table S2**. Annotation information of BW1.

**Additional file 3: Table S3**. Annotation information of BW3.

**Additional file 4: Table S4**. Functional annotation of genes.

**Additional file 5: Table S5**. KEGG pathway analysis.

## Data Availability

The chicken 60 k SNP data used in this article, including the additional files, have been deposited into Gene Expression Omnibus (http://www.ncbi.nlm.nih.gov/geo/) with the identifier GSE58551.

## Abbreviations

GO: Gene ontology
GWAS: Genome-wide association study
KEGG: Kyoto encyclopedia of genes and genomes
LD: Linkage disequilibrium
QTL: Quantitative trait loci
SNP: Single nucleotide polymorphism
