# Supplementary material for: A time-dependent genome-wide SNP-SNP interaction analysis of chicken body weight
Source: BMC Genomics. 2019 Oct 23;20:771. doi: 10.1186/s12864-019-6132-0 (PMC6813082; doi:10.1186/s12864-019-6132-0)
Supplement: Supplementary file 1 — Additional file 1: Table S1. Annotation information of BW0. [file 12864_2019_6132_MOESM1_ESM.pdf]

Table S1. Annotation information of BW0

GGA	SNPname	Region_start	Region_end	RefSeq Gene	microRNA
chr1	GgaluGA010224	28812405	29812405	*CNTN1, GXYLT1, YAF2, ZCRB1, PPHLN1*	*MIR7447*
chr1	GgaluGA016211	47454656	48454656	*ARL1, CNOT4, WDR91, PDE6H, ARHGDIB, MGP, OC3, ART4, HIST1H2B7, HIST1H46L2, HISTH2A4L1, HIST2H4B, HIST1H46, HIST1H2B7, HIST1H110, HIST1H46L2, HIST1H2B8, HIST2H3L, H2AFJ, HIST1H103, HIST1H2A4, HIST1H101, HIST1H111L, HIST1H2A4L3, HIST1H2B5, HIST1H3H, HIST1H111R, DDX47, HEBP1, FAM234B, EMP1*	*MIR6581*
chr1	Gga_rs10727935	48957471	49957471	*UBN2, LUC7L2, GBE, POLDIP3, RRP7A, SERHL2, SOCS1L, CYP2D6, NDUFA6, NAGA, WBP2NL, CENPM, TNFRSF13C, XRCC6, POLR3H, ACO2, PHF5A, TOB2, TEF, RANGAP1, CHADL, L3MBTL2, RBX1, ST13*	*MIR1581, MIR33A, MIR1683*
chr1	Gga_rs13982417	177968418	178968418	*FGF9, SAP18, XPO4, EEF1AKMT1, IL17D, CRYL1, GJB6*	
chr2	Gga_rs14135538	7722124	8722124	*INSIG1, EN2, SHH, LMBR1, MNX1, UBE3C, DNAJB6*	
chr2	Gga_rs14184594	54707646	55707646	*IGFBP1, IGFBP3, TNS3, SLC12A7*	
chr2	GgaluGA160608	101049357	102049357	*TGIF1, MYL12A, MYOM1, LPIN2, NDC80*	
chr3	Gga_rs14082553	15367488	16367488	*DTD1, SEC23B, DZANK1, BIRC5, POLR3F, KAT14, MGME1, SNX5, RRBP1, DSTN, CST3, CST7, APMAP, TTBK1, SLC22A7, TTL, VSX1, ENTPD6, MAL, MRPS5, SLC8A1*	*MIR3526*
chr3	GgaluGA210739	17890110	18890110	*AIDA, MIA3, TAF1A, DUSP10, MARC1, RAB3GAP2, IARS2, BPNT1, EPRS*	*MIR194-1, MIR215*
chr3	Gga_rs14323198	19504341	20504341	*GPATCH2, ESRRG*	
chr3	Gga_rs14385387	80891797	81891797	*TMEM30A, COX7A2, COL12A1, SLC17A5, EEF1A1, MTO1*	*MIR6687, MIR7465*
chr3	Gga_rs16320563<br>Gga_rs14394679<br>Gga_rs15426103<br>Gga_rs14395789	90372221	92888732	*MYOM2, CLN8, ERICH1, ACP1, TMEM18*	
chr4	Gga_rs15474576	2027564	3027564	*BTK, TIMM8A, TAF7L, CENPI, DKC1, MPP1, RHOGL, NONO, GJB1, NLGN3, IL2RG, SNX12, LOC422214, HTR2C, IL13RA2, PLS3*	*MIR222B, MIR460B,*
chr4	Gga_rs16424343	63055765	64055765	*ASAH1, PCM1, FGL1, SLC7A2, CNOT7*	*MIR1605*
chr4	Gga_rs14490998	75845663	76845663	*LCORL, LAP3, QDPR, LDB2*	*MIR1602*
chr4	Gga_rs14493884<br>GgaluGA267201	78970536	80719288	*TMEM128, NSG1, STX18, MSX1, EVC2, EVC, CRMP1, TBC1D14, GRPEL1*	*MIR1701*
chr4	Gga_rs13665914	81170167	82170167	*AFAP1, CPZ, SOHO-1, HMX1*	
chr4	GgaluGA268612	83708098	84708098	*LOC422894, NELFA, LETM1, FGFR3, TACC3, TMEM129, SLBP, FAM53A*	
chr5	GgaluGA289789	52943281	53943281	*HSPA2, MTHFD1, ESR2, WDR89, RHOJ, ERNI*	
chr5	Gga_rs15736571	53515011	54515011	*RHOJ, ERNI, HIF1A, SIX1, SIX6*	
chr6	GgaluGA295929	5974041	6974041	*PCDH15, SIRT1, DNAJC12, CTNNA3, LRRTM3*	
chr6	Gga_rs14588369	26695295	27695295	*TECTB, ACSL5, VTI1A, TCF7L2, HABP2, DCLRE1A, NHLRC2*	
chr7	Gga_rs10728585	12216783	13216783	*METTL21A, CREB1, KLF7, ADAM23, EEF1B2, NDUFS1, NRP2*	
chr7	Gga_rs14614638	20448903	21448903	*IFIH1, FAP, GCG, DPP4*	

chr7	Gga_rs15871969	26629442	27629442	*SEC22A, ADCY5, HACD2, MYLK*	
chr9	Gga_rs13608349	14029761	15029761	*P3H2, TP63, LPP, BCL6, SST, MASP1, SLC51A, TFR2*	*MIR1653*
chr11	GGaluGA078115	13179945	14179945	*NUDT7*	*MIR6595*
chr12	Gga_rs14031249<br>GgaluGA081258	1194961	2244994	*RFT1, PRKCD, NPRL2, CISH, MAPKAPK3*	
chr13	GgaluGA093775	8359804	9359804	*CSNK1A1L, STK10, DUSP1, RPL26L1, RPL26, NKX2-5*	*MIR6652, MIR3523*
chr13	GgaluGA096558	13575053	14575053	*HNRNPH2, DGUOK, RUFY1, MAPK9, RASGEF1C, HNRNPAB, NME5, WNT8A, FAM13B, NPY6R*	
chr14	Gga_rs15719971	1328328	2328328	*PDGFA, GET4, COX19, CYP2AC7, GPR146*	
chr19	GgaluGA124944<br>GgaluGA125308<br>Gga_rs15045504<br>Gga_rs15045732<br>Gga_rs14118327	1728331	3504813	*CASTOR2, RCC1L, NCF1, RFC2, LAT2, EIF4H, UBE2G1, ATP2A3, P2RX1, MIS12, RABEP1*	*MIR1354, MIR1587, MIR7444, MIR6592*
chr19	GgaluGA126270<br>Gga_rs14119969<br>Gga_rs10730456<br>Gga_rs15048206<br>Gga_rs15048223	3823581	5935922	*CUX1, PRKRIP1, ORAI3, RASA4, YWHAG, HSPB1, POR, RAD51D, RFFL, LIG3, CCLI5, CCL1, CCAH221, MRPS17, NIPSNAP2, PSPH, CCT6A, PHKG1, CHCHD2,VKORC1L1, GUSB, ASL2, ASL1, CRCP, KCTD7, NCBP3, CRK, YWHAE, MYO1C, INPP5K, SERPINF1, SMYD4, RPA1, DPH1, HIC1, POLDIP2, VTN, SLC46A1, ALDOC, TLCD1, FAM222B, ERAL1, FLOT2*	*MIR6585, MIR22, MIR1666, MIR1696, MIR451B, MIR144*
chr20	Gga_rs14277625	9127832	10127832	*EEF1A2, PPDPF, NPBWR2, OPRL1, RGS19, SOX18, ZNF512B, UCKL1, DNAJC5, TPD52L2, STMN3, SIRPA, NSFL1C, FKBP1A, PSMF1, SCRT2*	*MIR1798*
chr24	GgaluGA193154	4364494	5364494	*UBE4A, USP28, ZBTB16, RBM7, REXO2, BUD13, APOA4, APOC3, APOA1, PAFAH1B2, TAGLN, PCSK7*	*MIR6612, MIR6671, MIR1745-1, MIR1667*
chr27	GgaluGA199670	2587569	3587569	*ACE, KCNH6, DCAF7, LIMD2, RNF113A, STRADA, DDX42, MYL4, CDC27, MAPT, ITGA3, DLX3, KAT7, SLC35B1, NGFR, MEOX1, PHB*	
chr27	Gga_rs16208036	3939774	4939774	*MRPL45, CBX1, NFE2L1, CDK5RAP3, LOC107055293, PCGF2, RPL23, LASP1, RPL19, ERBB2, IKZF3, ZPBP2, GSDMA, PSMD3, CSF3, MED24, THRA, RARA, TOP2A, IGFBP4, CCR7, SMARCE1, KRT222, KRT20*	*MIR6663, MIR1735, MIR6547*
Chrz	Gga_rs15249625	4808255	5808255		
Chrz	Gga_rs16763798	15608595	16608595	*ITGA1, PELO, FST*	*MIR1631*
Chrz	Gga_rs14753903	19491662	20491662	*HTR1A*	
Chrz	Gga_rs14755141<br>Gga_rs16101791	20990644	22408798	*SLC30A5, CENPH, THBS4, MTX3*	
Chrz	Gga_rs16764637<br>Gga_rs14759170<br>Gga_rs14759127<br>GgaluGA349792<br>Gga_rs16764173	23312341	24848301	*S100Z, F2RL1, IQGAP2, POLK, HMGCR, NSA2, UTP15, ANKRA2, FOXD1*	
Chrz	Gga_rs16106712<br>Gga_rs16106786<br>Gga_rs16071074<br>Gga_rs16091907<br>Gga_rs16091913	27838607	32762749	*GLDC, TYRP1, MPDZ, NFIB, ZDHHC21, CER1, PSIP1*	*MIR1779*

	Gga_rs16065879				
	Gga_rs14738375				
	Gga_rs13816749				
	Gga_rs16080645				
	Gga_rs16106257				
	Gga_rs16685135				
	Gga_rs14691748				
	Gga_rs16776264				
	GgaluGA350520				
	Gga_rs14762941				
	Gga_rs16108466				
Chrz	Gga_rs14767978	51048433	52048433	*CHD1Z, RIOK2, LIX1, TRIM14, NANS, CLTA*	
	Gga_rs14775753				
chrz	Gga_rs16774940	65912281	67063604	*INIP, GNG10, SMC2, PTGR1, TXN, MuSK, LPAR1*	*MIR1452, MIR1459*
	Gga_rs16774954				
