# Supplementary material for: A time-dependent genome-wide SNP-SNP interaction analysis of chicken body weight
Source: BMC Genomics. 2019 Oct 23;20:771. doi: 10.1186/s12864-019-6132-0 (PMC6813082; doi:10.1186/s12864-019-6132-0)
Supplement: Supplementary file 2 — Additional file 2: Table S2. Annotation information of BW1. [file 12864_2019_6132_MOESM2_ESM.pdf]

Table S2. Annotation information of BW1

GGA	SNPname	Region_start	Region_end	RefSeq Gene	microRNA
chr1	GgaluGA061615	186472115	187472115	*FAT3*	
chr2	GgaluGA133839	12534583	13534583	*CREM, CUL2, PARD3*	
chr2	GgaluGA136764	20749825	21749825	*ABCB1, RUNDC3B, SRI*	
chr3	GgaluGA211595	21019480	22019480	*CENPF, SMYD2, PROX1, ANGEL2, NSL1*	*MIR1649*
chr4	Gga_rs14491923	76886283	77886283	*LDB2, TAPT1, PROM1, FGFBP2, CD38, BST1*	
chr5	Gga_rs13756660<br>Gga_rs14515483	11016916	12464976	*SOX6, C5H11orf58, RPS13, NUCB2, FTL, MYOD1, TPH1*	
