# Supplementary material for: A time-dependent genome-wide SNP-SNP interaction analysis of chicken body weight
Source: BMC Genomics. 2019 Oct 23;20:771. doi: 10.1186/s12864-019-6132-0 (PMC6813082; doi:10.1186/s12864-019-6132-0)
Supplement: Supplementary file 3 — Additional file 3: Table S3. Annotation information of BW3. [file 12864_2019_6132_MOESM3_ESM.pdf]

Table S3. Annotation information of BW3

GGA	SNPname	Region_start	Region_end	RefSeq Gene	microRNA
chr5	GgaluGA273676	7314530	8314530	*CYB5R2, FAR1, PTH, BTBD10, ARNTL, TEAD1, DKK3, USP47, GALNT18*	*MIR1568*
chr7	Gga_rs13598324	28189987	29189987	*SCTR, DBI, C7H2ORF76, MARCO, INSIG2, CCDC93*	
chr11	Gga_rs14965049	11871228	12871228	*CDH11*	
chr12	Gga_rs14045047	14335430	15335430	*SLC25A26, SUCLG2, EOGT*	
