# Supplementary material for: A time-dependent genome-wide SNP-SNP interaction analysis of chicken body weight
Source: BMC Genomics. 2019 Oct 23;20:771. doi: 10.1186/s12864-019-6132-0 (PMC6813082; doi:10.1186/s12864-019-6132-0)
Supplement: Supplementary file 4 — Additional file 4: Table S4. Functional annotation of genes. [file 12864_2019_6132_MOESM4_ESM.pdf]

Table S4. Functional annotation of genes

Term	Genes	Corrected p-value
GO:0044815 DNA_packaging_complex	HIST1H46L2, HIST1H46, H2AFJ, SMC2, HIST2H4B, HIST1H111L, HIST1H110, HIST1H101, HIST1H2B5, HIST1H2B8, HIST1H2A4, HIST1H2B7, HIST1H103, HIST1H2A4L3, HIST1H3H, HIST1H111R	3.02E-07
GO:0000786 nucleosome	HIST1H46L2,HIST1H46,H2AFJ,HIST2H4B,HIST1H111L,HIST1H110,HIST1H101,HIST1H2B5,HIST1H2B8,HIST1H2A4,HIST1H2B7,HIST1H103,HIST1H2A4L3,HIST1H3H,HIST1H111R	8.46E-07
GO:0032993 protein-DNA_complex	HIST1H46L2,HIST1H46,H2AFJ,TCF7L2,HIST2H4B,RPA1,HIST1H111L,HIST1H110,HIST1H101,HIST1H2B5,HIST1H2B8,HIST1H2A4,HIST1H2B7,HIST1H103,HIST1H2A4L3,HIST1H3H,HIST1H111R	5.12E-05
GO:0044427 chromosomal_part	RAD51D,HIST1H46L2,XRCC6,TP63,RANGAP1,CBX1,TCF7L2,HIST2H4B,RPA1,HIST1H111L,PCGF2,HIST1H101,HIST1H2A4,HIST1H103,PSIP1,BCL6,SOX18,TOP2A,HIST1H111R,CENPM,CREB1,HIST1H46,BIRC5,NDC80,H2AFJ,SIRT1,SMC2,CENPI,MIS12,CENPH,DCLRE1A,HIST1H110,SMARCE1,HIST1H2B5,RFC2,HIST1H2B8,HIST1H2B7,HIST1H2A4L3,HIST1H3H	1.63E-04
GO:0005694 chromosome	RAD51D,HIST1H46L2,XRCC6,TP63,RANGAP1,CBX1,TCF7L2,HIST2H4B,RPA1,HIST1H111L,PCGF2,HIST1H101,HIST1H2A4,HIST1H103,PSIP1,BCL6,SOX18,TOP2A,HIST1H111R,CENPM,CREB1,HIST1H46,BIRC5,NDC80,H2AFJ,SIRT1,SMC2,CENPI,MIS12,CENPH,DCLRE1A,HIST1H110,SMARCE1,HIST1H2B5,RFC2,HIST1H2B8,HIST1H2B7,HIST1H2A4L3,HIST1H3H	0.001168253
GO:0000785 chromatin	HIST1H46L2,TP63,CBX1,TCF7L2,HIST2H4B,HIST1H111L,PCGF2,HIST1H101,HIST1H103,HIST1H2A4,PSIP1,SOX18,TOP2A,HIST1H111R,CREB1,HIST1H46,H2AFJ,SIRT1,SMARCE1,HIST1H110,HIST1H2B5,HIST1H2B8,HIST1H2B7,HIST1H2A4L3,HIST1H3H	0.004101226
GO:0000228 nuclear_chromosome	RAD51D,HIST1H46L2,XRCC6,TP63,CBX1,TCF7L2,HIST2H4B,RPA1,PCGF2,HIST1H2A4,PSIP1,SOX18,TOP2A,CREB1,NDC80,HIST1H46,H2AFJ,SIRT1,SMC2,MIS12,DCLRE1A,SMARCE1,HIST1H2B5,HIST1H2B8,HIST1H2B7,HIST1H2A4L3	0.005742582
GO:0044454 nuclear_chromosome_part	RAD51D,HIST1H46L2,CREB1,XRCC6,TP63,HIST1H46,NDC80,CBX1,H2AFJ,SIRT1,TCF7L2,MIS12,HIST2H4B,RPA1,PCGF2,DCLRE1A,SMARCE1,HIST1H2B5,HIST1H2B8,HIST1H2A4,HIST1H2B7,PSIP1,HIST1H2A4L3,SOX18	0.025538444
