# Supplementary material for: A time-dependent genome-wide SNP-SNP interaction analysis of chicken body weight
Source: BMC Genomics. 2019 Oct 23;20:771. doi: 10.1186/s12864-019-6132-0 (PMC6813082; doi:10.1186/s12864-019-6132-0)
Supplement: Supplementary file 5 — Additional file 5: Table S5. KEGG pathway analysis. [file 12864_2019_6132_MOESM5_ESM.pdf]

Table S5. KEGG Pathway analysis

Term	ID	Corrected P-Value	Hyperlink
Calcium signaling pathway	gga04020	0.005	http://www.genome.jp/kegg-bin/show_pathway?gga04020/gga:396445%09red/gga:417543%09red/gga:395707%09red/gga:395190%09red/gga:426130%09red/gga:428707%09red/gga:417509%09red
Focal adhesion	gga04510	0.009	http://www.genome.jp/kegg-bin/show_pathway?gga04510/gga:396306%09red/gga:417553%09red/gga:426130%09red/gga:396445%09red/gga:373946%09red/gga:395935%09red/gga:395951%09red
ECM-receptor interaction	gga04512	0.021	http://www.genome.jp/kegg-bin/show_pathway?gga04512/gga:396306%09red/gga:395935%09red/gga:395951%09red/gga:373946%09red
Melanogenesis	gga04916	0.035	http://www.genome.jp/kegg-bin/show_pathway?gga04916/gga:395340%09red/gga:395913%09red/gga:395099%09red/gga:395508%09red
Oocyte meiosis	gga04114	0.038	http://www.genome.jp/kegg-bin/show_pathway?gga04114/gga:427820%09red/gga:395340%09red/gga:417554%09red/gga:419960%09red
